# Development and validation of an indirect ELISA as a confirmatory test for surveillance of infectious bovine rhinotracheitis in vaccinated herds

**DOI:** 10.1186/s12917-015-0612-5

**Published:** 2015-12-08

**Authors:** Luigi Bertolotti, Elvira Muratore, Chiara Nogarol, Claudio Caruso, Laura Lucchese, Margherita Profiti, Laura Anfossi, Loretta Masoero, Stefano Nardelli, Sergio Rosati

**Affiliations:** Department of Veterinary Science, University of Torino, Torino, Italy; Istituto Zooprofilattico Sperimentale di Piemonte, Liguria e Valle d’Aosta, Torino, Italy; Istituto Zooprofilattico Sperimentale delle Venezie, Legnaro, Padova, Italy; Department of Chemistry, University of Torino, Torino, Italy; Present address: Largo Paolo Braccini, 2, 10095 Grugliasco, TO Italy

**Keywords:** IBR, Indirect ELISA, Confirmatory test

## Abstract

**Background:**

*Bovine herpesvirus 1* (BoHV1) is a member of the viral subfamily of *Alphaherpesvirinae* that infects various species, including cattle, sheep, and goats. The virus causes infectious bovine rhinotracheitis (IBR), which is included in a European list of diseases that may require control and eradication programs. The lack of confirmatory tests affects the validity of diagnostic tools, especially those used for vaccinated herds. In this study, we report the development and validation of an indirect enzyme-linked immunosorbent assay (ELISA) based on BoHV1 glycoprotein E, which was expressed as a secreted recombinant antigen in a mammalian cell system. The performance of the new rec-gE ELISA was compared with that of commercially available indirect and/or blocking ELISAs.

**Results:**

The sample set included blood sera from animals from IBR-positive farms, IBR-free farms, and marker-vaccinated farms. The indirect ELISA proposed in this study is based on antibody reactivity against BoHV1 gE, and showed high sensitivity and specificity (98.41 and 99.76 %, respectively).

**Conclusions:**

The ELISA performed well, in terms of both its diagnostic sensitivity and specificity, and as a confirmatory methodology, and therefore should improve the diagnostic protocols used for IBR surveillance.

## Background

*Bovine herpesvirus 1* (BoHV1) is a member of the viral subfamily of *Alphaherpesvirinae* that infects different species, including cattle, sheep, and goats. The respiratory syndrome produced in cattle is known as infectious bovine rhinotracheitis (IBR), and genital infections are associated with pustular vulvovaginitis, balanoposthitis, and abortion. Both the respiratory syndrome and genital infection may cause lifelong latent infection. The virus is self-reactivating or is reactivated by stress [1] or treatment with corticosteroids [2], causing relapse. Although mortality is low, the disease has a severe impact on growth, milk production, and the international livestock trade, causing it to be included in a European list of diseases that may require control and eradication programs (64/432/CEE [3]).

The epidemiological situation varies across different countries [1]. In response to European Union (EU) regulations, several European countries have adopted different strategies for the eradication of IBR, and several countries have achieved an IBR-free status (Sweden, Austria, Denmark, Finland, Switzerland, Norway, the Federal State of Bavaria, and the Province of Bolzano in Italy) and have established trade restrictions for seropositive animals (2004/558/CEE [4]).

A glycoprotein E (gE)-negative strain of BoHV1 has been shown to be an effective and safe tool for IBR control [5] and reduces the shedding of viral particles [6, 7]. The use of DIVA (differentiation of infected from vaccinated animals) vaccines and planning surveillance measures [1, 8] should allow the serological differentiation of infected and vaccinated cattle [9–11].

Because the most common approach to BoHV1 control involves the use of gE-deleted vaccines [10, 12], serological studies are based on a combination of whole-virus-based indirect ELISA or gB-based blocking ELISA, together with a gE-based blocking ELISA. The gE ELISA approach includes blocking assays that were developed using a monoclonal antibody that recognizes a single conformational epitope present on the gI–gE complex in the wild-type virus [13]. However, it has been demonstrated that some gE-blocking ELISAs are not absolutely specific and sensitive [14]. In some cases, hypervaccinated animals can give false-positive results attributable to nonspecific blocking, caused by the steric hindrance afforded by the high antibody titers against other BoHV1 glycoproteins, such as gB, gC, gD, and gI [13, 15]. Such false-positive reactions can also occur when testing “fresh” sera because of a phenomenon that occurs in samples that are not frozen and heat-inactivated, as was suggested for the BoHV1 gE ELISAs [16]. In contrast, comparison of the gE ELISA with the highly sensitive gB ELISA [14] showed the 98 % relative sensitivity of the diagnostic protocol [13]. Reference sera were selected, so that competitive serological test procedures could be performed with stable references [17]. Despite the use of these sera in all validation processes, an evaluation of the diagnostic tests conducted in 2001 and published three years later suggested that the standardization and harmonization of the tests should be ongoing, and performed with increased numbers of reference sera [14]. To our knowledge, the only commercially available tests capable of discriminating between infected and vaccinated animals during immunization programs are blocking ELISAs. Diagnostic test performance is especially important during the last phases of eradication programs, and confirmatory tests are required to clarify possibly doubtful results. In this study, we developed and validated an indirect ELISA based on BoHV1 gE expressed as a secreted recombinant antigen in a mammalian cell system.

## Results and discussion

In total, 189 field sera were classified as positive with different commercial ELISAs, based on both indirect and blocking approaches. Of these, 186 were positive when tested with the rec-gE ELISA. A single serum was positive on the IDEXX Trachitest Serum Screening Ab Test and on IDEXX IBR gB and rec-gE ELISAs, but was negative on the IDEXX IBR gE Ab blocking ELISA test. All three positive samples that were incorrectly classified with the rec-gE ELISA were identified as positive with both the indirect and the gB and gE blocking ELISAs.

**Fig. 1:**
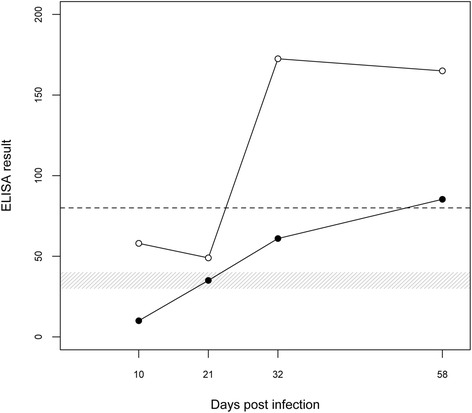
Seroconversion of experimentally infected animals. Sera were tested with the blocking ELISA (black circles) and the rec-gE indirect ELISA (white circles). The gray area shows the window of uncertainty in the interpretation of results for the blocking ELISA. The horizontal dashed line indicates the cut-off for the rec-gE ELISA

**Fig. 2:**
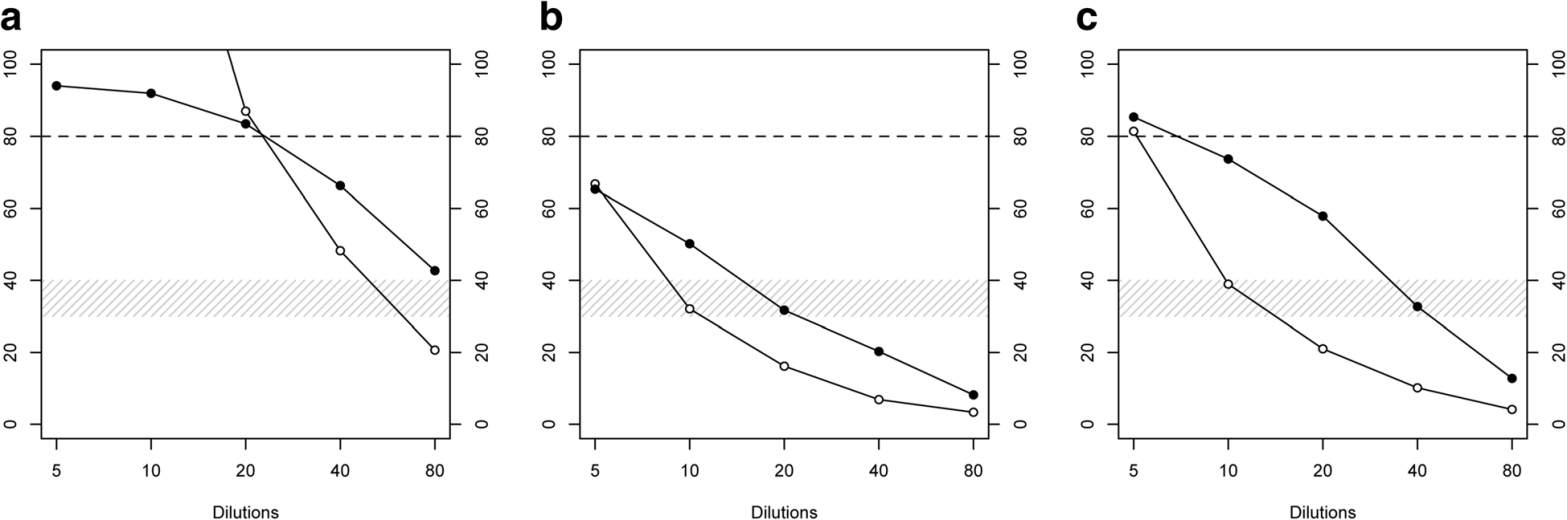
Analytical sensitivity. Field sera **a**, **b**, and **c** were serially diluted with negative serum and tested with the blocking ELISA (black circles) and the rec-gE indirect ELISA (white circles). The gray area shows the window of uncertainty in the interpretation of the results for the blocking ELISA. The optimal cut-off for the rec-gE ELISA should be reevaluated (see Discussion)

All seven sera collected after experimental infection were tested with both the IDEXX gE blocking ELISA and the rec-gE indirect ELISA. Each serum was diluted to test the analytical sensitivity. The rec-gE ELISA showed good precocity, classifying samples as positive 32 days after infection when diluted 1:4 (Fig. 1). Three different field sera were serially diluted with the same procedure before testing, to simulate different antibody titer scenarios. Clearly, the antibody titer strongly influenced the analytical sensitivity. The blocking ELISA identified the diluted sera as positive at higher dilutions than the rec-gE indirect ELISA in all cases (Fig. 2). The results obtained from pooled samples can be interpreted using different cut-off settings [18]. Therefore, we evaluated the performance of the new rec-gE ELISA on pooled blood serum samples, so that the cut-off level could be modified based on the pool size to improve its analytical sensitivity.

**Fig. 3:**
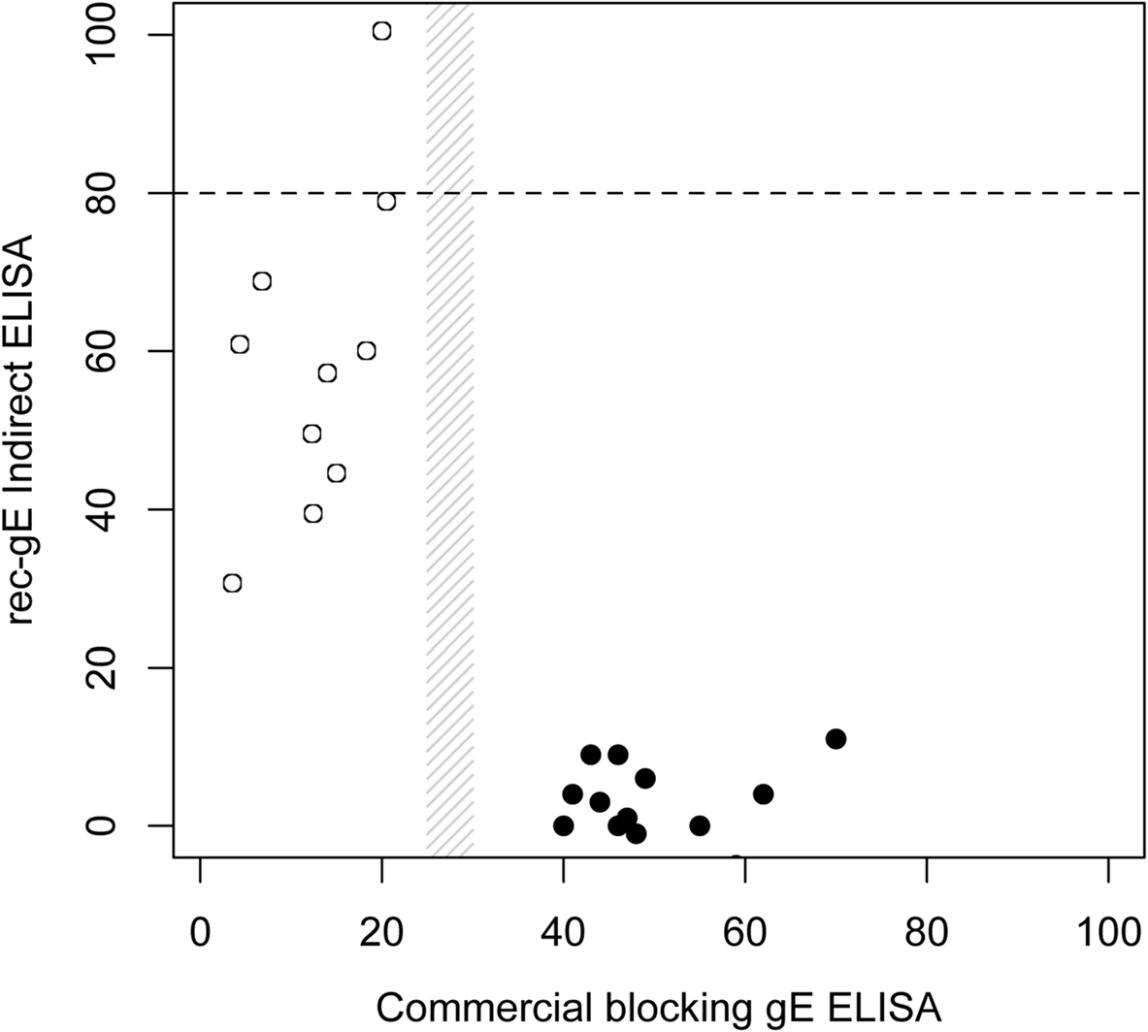
IBR-false-positive sera. Reactivity of sera showing false-positive results when tested with the blocking ELISA (black circles) and the rec-gE indirect ELISA (white circles). The gray area shows the window of uncertainty in the interpretation of the results for the blocking ELISA. The horizontal dashed line indicates the cut-off for the rec-gE ELISA

**Fig. 4:**
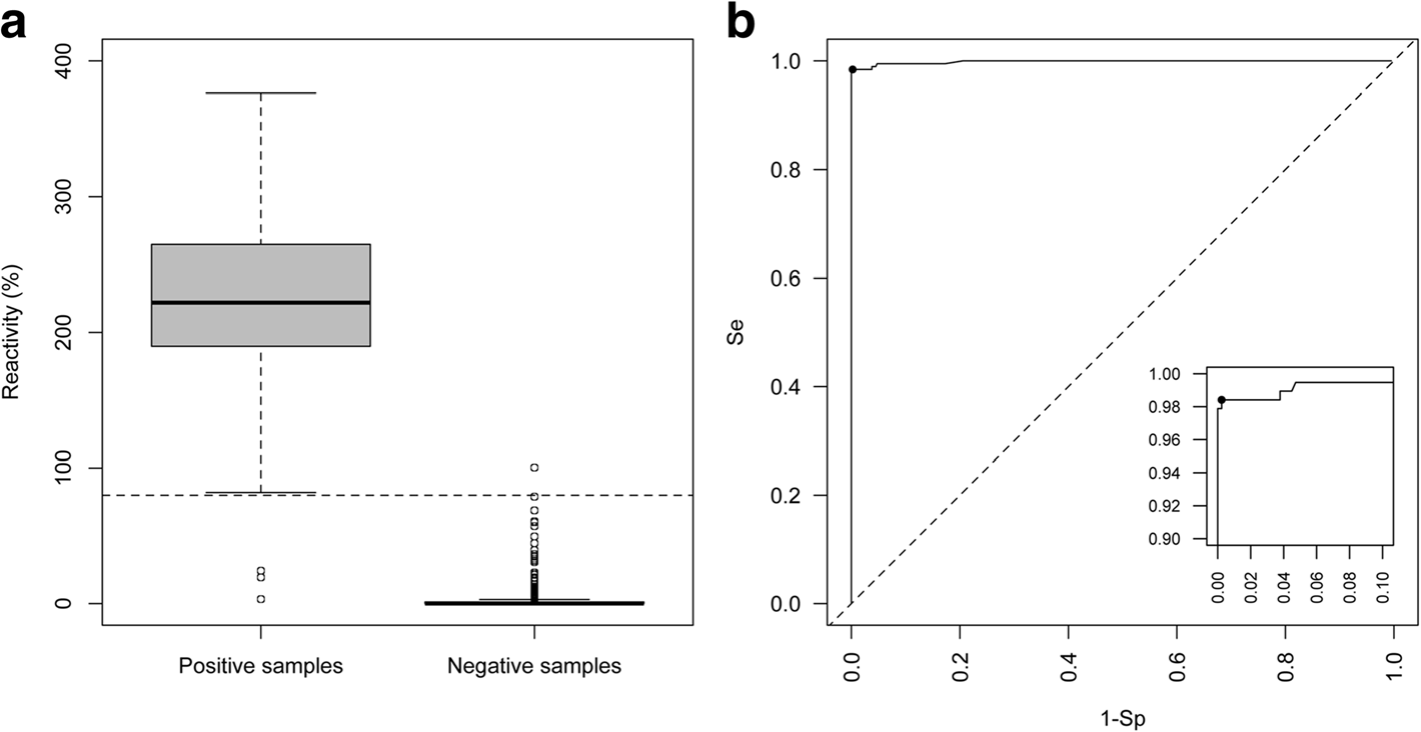
Results of the rec-gE ELISA. **a** Distribution of the reactivity of the tested sera; the horizontal dashed line indicates the cut-off for the rec-gE ELISA. **b** ROC curve showing the best sensitivity/specificity compromise (black dot). A magnification of the high-performance region is boxed within the graph

In total, 459 of 460 serum samples from gE-negative animals were negative on the rec-gE ELISA. Among the negative samples, a small subset (*n* = 16) showed reactivity when tested with the Hipra commercial gE blocking ELISA. All these samples were negative when tested with other commercial ELISAs (both indirect and blocking assays) or with the rec-gE ELISA. Only one serum from the IBR-free herd showed reactivity for the rec-gE ELISA, leading to a false-positive outcome. Interestingly, the false-positive results were different in the two tests, confirming the independence of the two approaches. The false-positive results in the two tests are shown in Fig. 3. The reactivity distribution of the sera tested with the rec-gE ELISA is shown in Fig. 4a. A receiver operating characteristic (ROC) curve analysis showed that the best cut-off value was 80 % of the positive control reactivity included in each plate (Fig. 4b). From this starting point, we calculated diagnostic sensitivity and specificity values of 98.41 % (95 % confidence interval [CI] 95.43–99.67 %) and 99.76 % (95 % CI 98.79–99.99 %), respectively.

In sequential testing, when positive results from the first analysis are assessed again with an independent test, the net effect is to increase the specificity and positive predictive value because each case is classified as positive on multiple tests (so false-positive results are rare). Therefore, an independent test should be used to confirm the positive results obtained with a blocking ELISA when screening herds, especially during the last phases of an eradication program. When the prevalence of the disease was low (less than 5 %), the proposed rec-gE ELISA achieved high positive and negative predictive values of 94.5 and 99.9 %, respectively. The rec-gE ELISA also seemed to show good precocity, similar to that of the commercial gE blocking ELISA.

The surveillance of IBR in IBR-free herds or regions is easily performed because there is a complete lack of antibodies against the viral proteins in unvaccinated, susceptible animals. If a wild-type viral strain should spread to the farm, the resulting massive immune response would be readily detectable. Whole-virus indirect ELISAs are commercially available and perform very well in the detection of BoHV1 antibodies. However, although vaccines confer obvious advantages in controlling viral spread within and among farms, vaccination programs can entail some pitfalls, especially during the last phases of an eradication program. In the last two decades, DIVA vaccines for IBR have been used as companion tests based on gE blocking ELISAs. The tests are very effective, but several factors must be considered. The most important involves confirmatory tests, which have been unavailable until now, but are essential during the last phases of an eradication program. It is well documented that the combination of gB and gE blocking ELISAs is an efficient way to control IBR on IBR-free and vaccinated farms [1, 19], but in both cases, some problems can arise. Blocking ELISAs based on reactivity against gB can suffer from less-than-absolute specificity when alphaherpesviruses genetically related to BoHV1 are circulating on the farm. For example, their partial cross-reactivity with antibodies directed against *Bovine herpesvirus 2* [18] or *Bubaline herpesvirus 1* [20] can result in IBR-positive outcomes on the gB blocking ELISA and lead to “vaccination-like” behavior. Consequently, the official procedures and restrictions prescribed by EU regulations should be applied in IBR-free regions (2004/558/CEE [4]).

The same scenario can occur on vaccinated farms, where a gE blocking ELISA can return false-positive results in hypervaccinated animals because of the steric hindrance of antibodies directed against viral proteins other than gE. Some sera from farms with a long history of immunization with DIVA vaccines and where the risk of viral circulation was very low produced positive results when tested with a commercial gE blocking ELISA. Further tests showed the true negative status of these samples, confirming the results obtained with the rec-gE indirect ELISA.

The indirect ELISA described in this study, based on the reactivity of IBR-infected animals to the gE protein only, performed well, and most importantly, seemed to be robust in the critical situations described above. No cross-reaction was observed with alphaherpesviruses that are genetically related to BoHV1 [20, 21] and no false-positive results were obtained in vaccinated herds.

It is interesting to note that rec-gE does not express the conformational epitope that is recognized by the monoclonal antibody used in the blocking ELISA, confirming that gI is required to detect diagnostic antibodies in blocking assays [21]. This strongly supports the independent nature of the rec-gE indirect ELISA. However, although gI is less immunogenic than other viral glycoproteins because of its proximity to gE, it may be responsible for potential false-positive reactions in blocking ELISAs when hypervaccinated animals are tested shortly after vaccination, which arises from steric hindrance.

The ectodomain of gE exposes different epitopes and this could explain its excellent sensitivity and diagnostic precocity. This aspect of the protein is particularly important in terms of herd surveillance, allowing the early diagnosis of new seroconversions that occur before the virus has spread rapidly within a farm. However, several points must be taken into account if a comparison between independent tests is to be made. Reference sera are one such important point. The sera used as references for blocking ELISAs (Perrin et al. 1994) may not be suitable for comparing the sensitivity of the rec-gE test with that of commercial blocking ELISAs because their reaction mechanisms are independent and different serum volumes are used in the two assays (50 μl in the blocking ELISAs and 10 μl in the rec-gE ELISA). Furthermore, the antibody titers seem to influence the sensitivity of this test because the rec-gE ELISA failed to identify diluted positive samples of field sera.

## Conclusions

Several European countries have implemented different surveillance and control programs to eradicate IBR, but the current economic insecurity has caused a reduction in the funds available to control infectious diseases in farm animals. Therefore, it is very important to improve the set of tools available, to identify the correct surveillance strategy, and to determine whether blood serum is the best diagnostic sample. Several studies have assessed the feasibility of using milk samples, especially from bulk milk, as a source of antibodies for IBR control. In IBR-free geographic areas, surveillance can be undertaken with commercially available tests, but in herds where DIVA vaccination is performed, blocking ELISAs can show low sensitivity and specificity. Further studies will evaluate the use of this rec-gE indirect ELISA in bulk milk testing.

## Methods

### Antigen preparation and ELISA procedure

To isolate the full-length BoHV1 gE gene, DNA was extracted from Madin–Darby bovine kidney (MDBK) cell cultures infected with a field strain of BoHV1 using a DNeasy Blood and Tissue Kit (Qiagen, Hilden, Germany). The DNA was used as the template in a PCR reaction. To clone and express the ectodomain of the protein a primer set was designed to bind downstream from the signal peptide (predicted with the SignalP4.0 software) and 14 residues upstream from the putative transmembrane domain (predicted with the Expasy TMpred tool). To facilitate directional cloning, each primer contained an appropriate restriction site (underlined) at the 5’ terminus (forward primer, *Bam*HI restriction site: 5’-TTGGATCCTAAGCCCGCGACCGAAACCC-3’; reverse primer, *Xho*I restriction site: 5’-TTCTCGAGTCTCGCTGGTGAGCGGTGGGC-3’). The LongRange PCR Kit (Qiagen) was used to amplify the targeted gene region, following the standard protocol proposed by the manufacturer. The amplified product of the expected length was column purified (NucleoSpin® Extract II Kit; Macherey-Nagel, Germany) and directly sequenced (BMR Genomics, Padua, Italy) using the PCR primers. The amplified gene fragment was digested with the appropriate restriction enzymes (Thermo Scientific) and ligated into the pSecTag2/Hygro plasmid (Invitrogen, USA), to allow the efficient intracellular sorting of the expressed protein and its secretion into the medium of transiently transfected mammalian cells. Ligation product was used to transform competent *Escherichia coli* (strain JM109) cells, and ampicillin-resistant colonies were rapidly screened with PCR and directly sequenced to confirm the authenticity and in-frame insertion of each fragment. The plasmid was purified from 25 ml of LB culture (~100 mg) using the Qiagen Plasmid Midi Kit. Subconfluent human embryonic kidney (HEK293T) cells, cultured in 75 cm^2^ flasks, were transfected with 6 ml of DMEM containing 9 μg of plasmid and 21 μl of Lipofectamine LTX Reagent (Invitrogen), according to a standard protocol [22]. After the cells were incubated for 6 h at 37 °C under 5 % CO_2_, the transfection medium was replaced with 6 ml of protein-free medium (EX CELL 293; Sigma-Aldrich) and the flasks were incubated as described above for a further 48 h. The medium was collected, centrifuged at 3000 × g for 10 min to remove the cell debris, and stored at –80 °C until analysis. Because the protein was expressed as a fusion protein, with a 6 × His tail, the protein concentration was roughly estimated using serial twofold dilutions in an indirect ELISA test coating of each supernatant into wells and probed with an anti-6 × His monoclonal antibody. A known amount of serially diluted recombinant 6 × His-tail-fused protein was used as the positive control to generate a standard curve, as previously described [21]. The complete sequence of the PCR fragment included 1158 bp of the gE ectodomain sequence, equivalent to 386 amino acids (patent pending: IT TO2014A000366). Protein expression was evaluated using a monoclonal antibody directed against the 6 × His tail. A 1:10 dilution of the cell supernatant was optimal for the test optimization (data not shown). The recombinant BoHV1 gE protein (medium from transfected cultures, even wells) or negative antigen (conditioned medium from untransfected cultures, odd wells) were diluted 1:10 in 0.1 M carbonate/bicarbonate buffer (pH 9.6) and used to coat the wells of a Nunc Maxisorp plate overnight at 4 °C. After the wells were blocked with 2.5 % bovine casein, the serum samples diluted 1:20 in phosphate-buffered saline (PBS)/1.25 % casein were added to the wells and the plates were incubated for 1 h at room temperature. After a washing step, peroxidase-labeled protein G, diluted to 10 ng/ml in PBS/1.25 % casein, was added and the plates were incubated for 45 min at room temperature. After the final wash, the color reaction was developed with 3,3′,5,5’-tetramethylbenzidine and stopped with 0.2 M H_2_SO_4_. The results were calculated as the percentage of serum reactivity compared with the positive controls (S/P) included in each plate. The cut-off value was determined by plotting the ROC curve and identifying the best sensitivity/specificity performance based on the reactivity of 189 gE-positive sera and 423 gE-negative sera. The ROC curve was drawn with the ROCR package of the R statistical software [23, 24].

### Serum samples

Different sets of blood serum samples from cattle were included in this study, collected from the Italian regions of Piedmont and Veneto. Among the samples, the field sera were collected during official surveillance procedures (*n* = 189), and the sera from experimentally infected animals (*n* = 7) were collected at different times and tested. Experimental infection was included in project no. 5885, on 09/07/2009, approved by the Ethics Committee of the Istituto Zooprofialttico Sperimentale delle Venezie according to article 7 of Decree No. 116, dated 27th January 1992.

All these sera were classified as positive based on the results of a commercially available whole-virus indirect ELISA (SVANOVA Svanovir IBR-Ab, IDEXX Trachitest Serum Screening Ab Test) and gB and/or gE blocking ELISAs (IDEXX IBR gE ab and gB Ab tests) . To evaluate analytical sensitivity, a small subset of sera from the field collection (*n* = 3) and all the sera from the experimentally infected animals were also serially diluted (twofold) in negative serum, and tested with the procedure described above.

The gE-negative serum set included 460 samples from both marker-vaccinated (*n* = 33) and IBR-free cattle (*n* = 427). All the sera from IBR-free cattle were negative when tested with two different commercially available gE blocking ELISAs and were expected to be negative on the rec-gE indirect ELISA. A subset of negative sera (*n* = 16) was selected because they generated false-positive outcomes when tested with a commercially available gE blocking ELISA. The true negative status of this subset was confirmed by repeating the sample collection and ELISAs (both blocking and whole-antigen indirect ELISAs), and epidemiological investigations at the herd level. This subset of samples was used to verify the independence of the proposed rec-gE indirect ELISA relative to the diagnostic blocking ELISA.

## Abbreviations

BoHV1: *Bovine herpesvirus 1*
IBR: Infectious bovine rhinotracheitis
DIVA: Differentiation infected from vaccinated animals
ELISA: Enzyme-linked immunosorbent assay
g*X*: Glycoprotein *X*
ROC curve: Receiver operating characteristic curve
MDBK: Madin–Darby bovine kidney
DMEM: Dulbecco’s modified Eagle’s medium
PBS: Phosphate-buffered saline
